# Epigenetics of Endometrial Cancer: The Role of Chromatin Modifications and Medicolegal Implications

**DOI:** 10.3390/ijms26157306

**Published:** 2025-07-29

**Authors:** Roberto Piergentili, Enrico Marinelli, Lina De Paola, Gaspare Cucinella, Valentina Billone, Simona Zaami, Giuseppe Gullo

**Affiliations:** 1 Institute of Molecular Biology and Pathology, Italian National Research Council (CNR-IBPM), 00185 Rome, Italy; roberto.piergentili@cnr.it; 2 Department of Medico-Surgical Sciences and Biotechnologies, Sapienza University of Rome, 04100 Latina, Italy; 3 Department of Anatomical, Histological, Forensic and Orthopedic Sciences, Sapienza University of Rome, 00161 Rome, Italy; 4 Department of Obstetrics and Gynaecology, AOOR Villa Sofia–Cervello, University of Palermo, 90133 Palermo, Italygullogiuseppe@libero.it (G.G.)

**Keywords:** endometrial cancer, chromatin modification, epigenetics, medical responsibility

## Abstract

Endometrial cancer (EC) is the most common gynecological malignancy in developed countries. Risk factors for EC include metabolic alterations (obesity, metabolic syndrome, insulin resistance), hormonal imbalance, age at menopause, reproductive factors, and inherited conditions, such as Lynch syndrome. For the inherited forms, several genes had been implicated in EC occurrence and development, such as *POLE*, *MLH1*, *TP53*, *PTEN*, *PIK3CA*, *PIK3R1*, *CTNNB1*, *ARID1A*, *PPP2R1A*, and *FBXW7*, all mutated at high frequency in EC patients. However, gene function impairment is not necessarily caused by mutations in the coding sequence of these and other genes. Gene function alteration may also occur through post-transcriptional control of messenger RNA translation, frequently caused by microRNA action, but transcriptional impairment also has a profound impact. Here, we review how chromatin modifications change the expression of genes whose impaired function is directly related to EC etiopathogenesis. Chromatin modification plays a central role in EC. The modification of chromatin structure alters the accessibility of genes to transcription factors and other regulatory proteins, thus altering the intracellular protein amount. Thus, DNA structural alterations may impair gene function as profoundly as mutations in the coding sequences. Hence, its central importance is in the diagnostic and prognostic evaluation of EC patients, with the caveat that chromatin alteration is often difficult to identify and needs investigations that are specific and not broadly used in common clinical practice. The different phases of the healthy endometrium menstrual cycle are characterized by differential gene expression, which, in turn, is also regulated through epigenetic mechanisms involving DNA methylation, histone post-translational modifications, and non-coding RNA action. From a medicolegal and policy-making perspective, the implications of using epigenetics in cancer care are briefly explored as well. Epigenetics in endometrial cancer is not only a topic of biomedical interest but also a crossroads between science, ethics, law, and public health, requiring integrated approaches and careful regulation.

## 1. Introduction

Endometrial cancer (EC) is the most common gynecological malignancy in industrialized countries and ranks as the fifth most frequent cancer in women [1]. Although its incidence increases with age and it typically occurs in postmenopausal women, a significant proportion of cases (15–25%) arises during reproductive age, when childbearing desires may still be present [2,3]. In these patients, the most common presenting symptoms include abnormal uterine bleeding and intermenstrual spotting, prompting thorough clinical evaluation through transvaginal ultrasound and, if indicated, diagnostic hysteroscopy. Histological confirmation, ideally via hysteroscopy-guided endometrial biopsy, is essential for establishing diagnosis and tumor grading [1].

In carefully selected young women with a desire for fertility, conservative management may be considered [4]. This approach is reserved for patients with endometrioid type, grade 1 (G1), stage IA EC confined to the endometrium, without evidence of myometrial invasion, lymphovascular space involvement, or adnexal spread [5,6]. Prior to initiating conservative treatment, comprehensive radiological assessment and operative hysteroscopy are crucial to rule out more advanced disease [7,8,9].

Fertility-sparing treatment usually involves hormonal therapy with progestins, such as medroxyprogesterone acetate (MPA) or megestrol acetate (MA), administered systemically or through levonorgestrel-releasing intrauterine devices (LNG-IUDs) [10]. Recent evidence suggests that combining hysteroscopic tumor resection with hormonal therapy improves complete response rates and reproductive outcomes compared to hormonal therapy alone [11,12,13]. It is also important to consider that many women eligible for fertility-sparing approaches present with comorbid conditions, such as obesity or polycystic ovary syndrome, that may independently contribute to infertility and increase the likelihood of requiring assisted reproductive technologies (ART) [12,14,15,16]. While the primary goal remains oncologic control, preserving reproductive potential in appropriately selected cases has become a feasible clinical strategy, supported by precise case selection, close monitoring, and multidisciplinary management. The 2021 ESGO/ESTRO/ESP molecular classification has further enhanced the selection process by identifying molecular subtypes associated with different risks of recurrence and prognosis [1]. For instance, mutations in POLE and PTEN are associated with favorable outcomes and may support conservative treatment, whereas alterations in p53, L1CAM, HER2, and FGFR2 are linked to poor prognosis, advising caution against fertility preservation strategies [4].

Importantly, beyond genetic mutations, growing evidence highlights the pivotal role of epigenetic mechanisms, particularly chromatin modifications, in the pathogenesis and progression of endometrial cancer. Chromatin refers to the dynamic complex of DNA and histone proteins that package genetic material within the nucleus. Chromatin structure modulates gene expression by regulating the accessibility of DNA to transcription factors and transcriptional machinery. Post-translational modifications of histone tails, such as acetylation, methylation, phosphorylation, and ubiquitination, can either condense chromatin into an inactive form (heterochromatin) or relax it into a transcriptionally active state (euchromatin), thereby promoting or silencing specific gene expression. These chromatin modifications are reversible and tightly regulated, playing a crucial role in maintaining endometrial homeostasis throughout the menstrual cycle [17,18]. Dysregulation of these epigenetic marks can result in abnormal activation or suppression of genes involved in cell proliferation, differentiation, apoptosis, and DNA repair mechanisms fundamental in tumor initiation and progression. Therefore, understanding chromatin alterations in EC not only provides insights into tumor biology but also opens new avenues for prognostic assessment and targeted epigenetic therapies [19].

In this context, new therapeutic and preventive perspectives are also emerging from nutraceutical and metabolic approaches. Molecules such as tea polyphenols and D-chiro-inositol, which have antioxidant, anti-inflammatory, and hormonal regulatory properties, are under investigation as potential adjuvant strategies in patients with endometrial cancer [20,21,22,23,24,25,26].

The article aims to examine the epigenetic landscape of endometrial cancer, with a specific focus on chromatin modifications. The main epigenetic marks, chromatin-modifying enzymes, and their interactions with oncogenic signaling pathways will be analyzed, with the goal of elucidating their contribution to endometrial tumorigenesis and their potential use as biomarkers or therapeutic targets.

## 2. Results

### 2.1. Molecular Bases of Chromatin Modification in Endometrial Cancer

#### Driver and Passenger Mutations

Tumorigenesis is the result of increasing genome instability causing the accumulation of mutations in genes that are essential for cell biology. Due to the high heterogeneity of tissue and cell types and to the different gene activation programs in different human body districts, each tumor shows a different molecular profile, which reflects not only the cell/tissue of origin but also the interaction of the tumoral cell with the surrounding environment (including the interaction with exogenous as well as endogenous stimuli) and the stochastic gene function alteration. On the one hand, this might be considered an advantage for oncologists, since it is possible to distinguish different cancer subtypes, identify the origin of metastases, make appropriate decisions about intervention, and, in the future, tailor adequate therapeutic strategies based on the molecular profile of each patient. However, this also implies that a general classification of tumors is nearly impossible, that cancer progression and response to therapy are hardly predictable, and that cytologically similar tumors may hide profound differences, impairing some therapeutic approaches such as chemotherapy. Gene mutations are directly related to genome instability; instability causes mutations, and mutations enhance instability, thus creating a reciprocal enhancement of DNA damage and malfunction. These events are selected by the tumor microenvironment [27,28,29], making the tumor “adapted” to new situations occurring over time that may span hypoxia, energy shortage, angiogenesis, apoptosis, inflammation, escape to immune surveillance, drug resistance, and others. Genome instability includes chromosome aberrations, aneuploidy, microsatellite instability, and increased nucleotide mutation rate [30,31].

Gene mutations in cancer are broadly divided into driver and passenger mutations [32]. Driver mutations are directly responsible for the behavior of cancer cells and affect central functions such as cell growth and division, apoptosis, DNA repair, and drug resistance. Thus, they are causally involved in oncogenesis since they grant a growth advantage to cancer cells; for this reason, they are positively selected in the cancer microenvironment. Passenger mutations are generally considered a byproduct of genome instability consequent to neoplastic transformation and may behave neutrally or even impair cancer cell survival to some extent; thus, they do not contribute to tumorigenesis. Indeed, some authors explain the efficiency of radio- and chemotherapy also through the accumulation of passenger mutations inside cancer cells, driving them to death after the cell genetic load increased over a defined threshold [33] that varies depending on the strength and role of the passenger mutations themselves [34]. It is important to emphasize here the difference between driver genes and driver mutations: Driver genes are those that are pivotal for cancer formation, progression, or treatment resistance; however, not all mutations in a driver gene can be classified as driver mutations. For example, in colorectal cancer, mutations in APC (a driver gene) are drivers only if they cause a truncation of the N-terminal part of the protein; otherwise (e.g., missense mutations), they are mostly considered passenger mutations [32]. Among the many mutations detectable in a tumor sample, only a minority of them are considered drivers. Some reports estimate that, on average, 40–100 mutations are present in cancer cells, of which only 5–15 are driver mutations [35,36,37]. Watanabe and collaborators analyzed 50 tumor-related genes in 100 surgical samples, finding that 8 genes are mutated at high frequency, namely *PTEN* (57%), *PIK3CA* (51%), *TP53* (30%), *KRAS* (23%), *CTNNB1* (21%), *FBFR2* (13%), *FBXW7* (10%), and *RB1* (9%), identifying them as EC driver genes [38]. These results are in good agreement with those reported earlier by Levine and collaborators [39], who analyzed 373 endometrial carcinomas and found extensive copy number alterations, few DNA methylation changes, low estrogen receptor/progesterone receptor levels, and frequent *POLE*, *TP53*, *PTEN*, *CTNNB1*, *PIK3CA*, *ARID1A*, and *KRAS* mutations, plus novel mutations in the chromatin remodeling complex gene *ARID5B* (see also below).

Bailey and collaborators, using a bioinformatics approach and analyzing the PanCancer and PanSoftware databases spanning 9423 tumor exomes and using 26 computational tools to catalogue driver genes and mutations, identified 299 driver genes, limited by focus on point mutations and small indels without consideration of copy-number variations, genomic fusions, or methylation events, and a total of over 3400 predicted driver mutations affecting 24 pathways/biological processes that drive tumor progression [40]. Notably, of the 299 driver genes identified, 18 genes are tagged as “chromatin histone modifiers” or “histone modification”, 14 as “chromatin other”, 8 as “chromatin SWI/SNF complex”, and 1 as “epigenetics DNA modifiers”; notably, out of these 41 genes, 13 (ca. 31.7%) had been identified in samples from uterine corpus endometrial carcinoma (UCEC) (Table 1).

In a recent publication, Stan and collaborators used artificial intelligence (AI) algorithms to identify driver mutations and genomic signatures in endometrial cancers [41]. The authors analyzed data from the COSMIC (Catalogue of Somatic Mutations in Cancers) database and found that approximately 2.5% of all mutations are drivers and cause cellular transformation and immortalization; moreover, endometrial cancers show distinct nucleotide substitution and chromosomal rearrangement signatures compared to other cancers.

**Figure 1:**
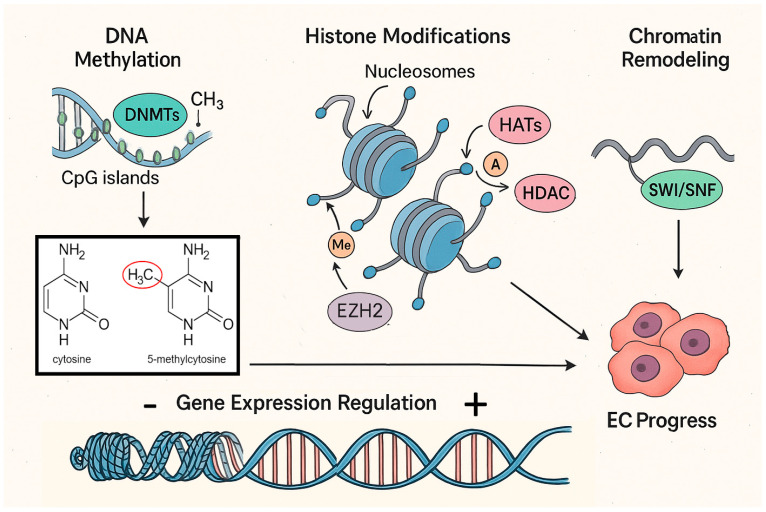
Graphical representation of the main events leading to DNA structure alteration in EC. Gene expression may either be enhanced or silenced through chromatin compaction regulation (bottom), which may be achieved by DNA methylation (top left), histone modifications (top center), or chromatin remodeling (top right); alteration of the expression of key genes may induce or enhance EC formation or progression. See text for further explanations and protein abbreviations. Additional abbreviations: Me: Methyl group; A: Acetyl group.

A sketch of the main chromatin modifications discussed in the next sections is reported in Figure 1.

### 2.2. Role of DNA Methylation in EC

“CpG islands” is the common name assigned to stretches of cytosine-guanine dinucleotides along DNA. These sequences represent the main sites of DNA methylation, that is, the addition of a methyl group to the deoxycytosine within the dinucleotide group; the reaction is catalyzed by DNA methyltransferases (DNMTs), and the human genome harbors five types of DNMTs: DNMT1, DNMT2, DNMT3a, DNMT3b, and DNMT3L [42]. The removal of methyl groups is due to the action of demethylases. Both DNMT1 and DNMT3B are overexpressed in type I EC but downregulated in type II EC [43,44]; as a consequence, hypermethylation of target genes (such as *PTEN*, *RASSF1*, *HAND2*, or *MLH1*) is more frequent in type I EC than in type II EC [17]. According to Edwards and collaborators [45], the human genome contains about 28 million CpG sites, ca. 60% of which have methylated cytosines. This epigenetic phenomenon causes transcriptional repression since methylated CpG islands recruit inhibitory proteins or prevent the interaction of transcription factors with DNA sequences. Consequently, the hypermethylation of DNA encoding tumor suppressor genes is directly linked to cancer development. Indeed, more than 50 promoters of tumor suppressor genes have been identified as hypermethylated in EC [46,47]. On the other hand, hypomethylation of DNA encoding proto-oncogenes may drive their overexpression or cause genome instability through various mechanisms, such as mutagenic transposable element mobilization [48]. For these reasons, several genes involved in this epigenetic gene expression control are considered cancer driver genes (Table 1).

Despite the large contribution of these modifications in cancer etiopathogenesis [49,50], in the works of both Levine and collaborators (Cancer Genome Atlas Research Network, 2013) and Bailey and collaborators [40], DNA methylation apparently seems to represent a minor feature of EC, with the former finding only a few DNA methylation alterations and the latter finding that just 13.7% (41/299) (Table 1) of driver genes control chromatin structure. Actually, reality is quite different. Wong and collaborators found hypermethylation of the *p16INK4A* gene promoter in 20% of EC samples [51]. Whitcomb and collaborators found high rates of methylation in the *HOXA11* and *THBS2* promoter regions; in addition, *HOXA11* promoter methylation was significantly more frequent in recurrent than non-recurrent cases and associated with poor outcome in early-stage EC [52]. Kang and collaborators found a significantly increased hypermethylation of *RASSF1A* in EC [53]. Notably, Yeh and collaborators found *PER1* (*period-1*) promoter hypermethylation in 31.4% of EC samples analyzed, suggesting a role also for circadian rhythms in EC development [54].

Aberrant DNA methylation is also associated with microsatellite instability (MSI) in 17–25% of EC, a phenotype correlated with defective DNA mismatch repair, a cause of genomic instability, and associated with hypermethylation of the *MLH1* promoter region in 71% of MSI+ samples analyzed [55]. Moreover, MSI+ EC is strongly associated with promoter hypermethylation of several genes, such as *hMLH1*, *PTEN*, *APC*, *SFRP1*, *SFRP4*, *SESN3*, *TITF1*, and *RASSF1A* [56,57,58,59,60]. Correlations between DNA methylation and MSI also involve other genes. *RASSF1A* methylation and *KRAS*/*BRAF* mutations were found only in MSI+ EC, and apparently *RASSF1A* methylation and *KRAS*/*BRAF* mutations are mutually exclusive in MSI- EC [53]. In many MSI+ EC, *SFRP1* also shows a hypermethylated promoter [61]. Moreover, the development and progression of type I endometrioid adenocarcinomas depend on the normal function of estrogen and progesterone receptors (ER and PR, respectively); it has been repeatedly shown that silencing of both *ER* and *PR* by aberrant DNA methylation is a recurrent feature in EC (summarized by [62]). Other hypermethylated genes include *p16*, *E-cadherin*, *CDH13*, *ESR1*, *O6-MGMT*, *PRs*, and *RARβ2* [18,63].

Instead, studies on hypomethylation in EC are relatively rare. To date, only a small number of hypomethylated genes have been identified, including *BMP* (bone morphogenetic protein), *CTCFL* (CCCTC-binding factor-like protein), *PARP1* (poly (ADP-ribose) polymerase 1), *CASP8* (caspase-8), *PAX2* (paired box 2), *NCAPH* (non-SMC condensin I complex subunit H), and *MCM* (minichromosome maintenance) ([63] and references therein). The role of hypomethylated ribosomal DNA genes is still under investigation, although this feature seems to characterize the prognosis of specific populations [64].

### 2.3. Histone Modifications and Chromatin Remodeling in EC

Chromatin compaction can also be regulated through histone modifications. DNA is wrapped around a nucleosome, an octamer of proteins (histones) called H2A, H2B, H3, and H4 (two of each type in the octamer). Histones are positively charged, and this favors the wrapping, DNA being negatively charged in its backbone. The positive histone charge is due to their amino acid composition, especially at their N-terminal end. There are several ways to modify these proteins, each having a different effect on the target, on the basis not only of the modification itself but also of its position along the histone sequence, total amount of modifications, and combination of different modifications, which lead researchers to define the so-called “histone code” [65]. Although most literature is focused on acetylation, methylation, and phosphorylation, several other modifications have been identified over time, including citrullination, ubiquitination, ADP-ribosylation, deamination, formylation, *O*-GlcNAcylation, propionylation, butyrylation, crotonylation, hydroxylation, and proline isomerization [66]. The most studied modification is lysine acetylation. In normally active genes, the ε-amines of lysines within the N-terminal ends of the core histones (H2A, H2B, H3, and H4) may be highly acetylated. Acetyl groups neutralize the basic charge at unmodified lysine residues, weakening the electrostatic interaction between negatively charged DNA and histones, releasing chromatin structure, and granting gene promoter access to the transcription machinery. However, some authors also hypothesize the role of histone acetylation in varying intracellular pH, a feature of neoplastic transformation [67]. Acetylation occurs through specialized “writer” proteins called histone acetyltransferases (HATs), while “erasers” are called histone deacetylases (HDACs); “readers” are a heterogeneous group of proteins characterized by specific motifs (such as chromodomain, bromodomain, or PHD finger) that have the role of recognizing such chromatin modifications and recruiting appropriate proteins to regulate gene expression accordingly (Table 2). In general, hypoacetylated histones strongly correlate with gene silencing.

Several proteins control histone (de)acetylation in EC. Among HDACs, there are HDAC1/2/3/4 and SIRT1/2/3/4/5/6/7; as for HATs, we recall here ELP3, p300, NCOA1/2/3, and MGEA5 [68]. However, it is worth recalling here that HAT/HDAC targets are not limited to histones but also involve other proteins central to EC development. Studies link HATs function to the regulation of steroid nuclear receptor genes [69], while other targets include estrogen receptors (ER), β-catenin, and TP53 [68].

Histone methylation is correlated to gene expression control as well. Histones may contain different levels of methylation: mono-, di-, and tri-methylation (me1, me2, and me3, respectively). In addition, arginine may be mono-methylated or di-methylated, the latter form being either symmetrical or asymmetrical [70]. Di- and trimethylation of histone H3 lysine 9 (H3K9me2/3), di-trimethylation of H3K27 (H3K27me2/3), and H4K20me3 are strongly associated with gene silencing, while trimethylation of histones H3 lysine 4 (H3K4me2/3), H3K27me1, H3K9me1, H4K20me1, and H3K36me1 are well-established gene-activating markers. This makes histone methylation a more subtle modification of chromatin, with contrasting effects depending on the site of modification [66], due to the absence of a net electrical charge. Also, in this case, methylase can act not only on histones but also on other target proteins. The main studied HMT in EC is EZH2, the major enzyme that methylates H3K27 residue [68]. Finally, other histone residues may undergo modifications—serine or threonine phosphorylation is an important posttranslational modification involved in the cellular response to DNA damage. For example, the histone variation H2AX is phosphorylated at Ser139 shortly after DNA damage, delimiting the region of chromatin around the DNA lesion [71]; the role of this histone variant in EC is well established [72,73].

There are four main families of chromatin-remodeling complexes: The switching defective/sucrose nonfermenting family (SWI/SNF), the imitation-switch family (ISWI), the Mi-2/nucleosome remodeling and histone deacetylation (NuRD) family (Mi2/Nurd), and the inositol 80 family (INO80/SWR1) [74]. All these proteins contain specific, characteristic domains (Table 2) and use ATP hydrolysis to modify DNA/histone interactions [75,76]. In EC, some of these chromatin modifiers are recurrently mutated (Table 3) such as MBD3 and MTA1, belonging to the Mi2/NuRD family; SMARCA2 (absent in Table 1), SMARCA4, and SMARCB1 (present in Table 1 but not linked to EC in [40]), belonging to the SWI/SNF family; and BRD4, which belongs to the BET (bromodomain and extra-terminal domain) family of proteins (reviewed in [68]).

### 2.4. Using Chromatin Modifications as Diagnostic, Prognostic, and Therapeutic Tools in EC

**Figure 2:**
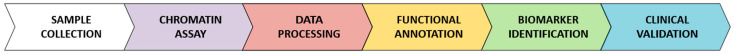
A simplified workflow illustrating the main steps necessary for the identification of candidate biomarkers in the EC.

“Omics” approaches can be efficiently used to identify candidate biomarkers useful for the clinical evaluation of patients; a simplified workflow for their identification is depicted in Figure 2.

DNA methylation detection may be performed in several ways, many of which involve minimally invasive approaches. Most available tests are aimed at finding hypermethylation of specific genes, because hypomethylation tests are less sensitive. The advantages of analyzing DNA methylation include the more stable nature of this change even after cell destruction or heavy sample manipulation and its early appearance during tumor development [77,78]. Sources of useful samples for this analysis include cervical scraping, vaginal fluid, urine, blood, and endometrial tissue from biopsies [70,71]. Once obtained, samples can be analyzed using diverse techniques, including—but not limited to—NGS; HPLC; HPCE; mass spectrometry; ELISA; and nanopore sequencing [48,79,80]. A recent systematic review and meta-analysis allowed the identification of a total of 31 genes from 20 studies as methylation markers in cytologic specimens and a total of 19 methylation markers from 10 studies for EC detection [81]. The study of the RAGE (receptor for advanced glycation end-products) signaling pathway in EC is at the beginning; thus, additional studies are required to verify if these pathways are under epigenetic control, particularly DNA methylation [82].

The analysis of the methylation status of specific genes allows us to predict, to some extent, the possible outcome of the disease and, in some cases, design an appropriate therapy. For example, the hypermethylation of the *MLH1* gene promoter or deficiencies in the expression of genes involved in DNA mismatch repair are affordable indicators of bad prognosis, higher relapse rates, and poor disease-specific survival [83,84,85]. Several other genes had been identified over the years, linking DNA methylation alteration and EC etiopathogenesis, some being validated experimentally, such as *E-cadherin* [86], *TESTIN* [87], *PTEN* [56], *TBX2*, *CHST11*, and *NID2* [19]. In addition, other hypermethylated genes were identified mainly on a bioinformatics basis, such as *MESDC1*, *LRRTM1*, *ATP8A2*, *OTX1*, *RYR1*, *NOVA1*, *C5orf38*, *IRX2*, *C9orf135*, *KCNQ2*, *SEPHS2*, and *DLGAP2* [88]; *CDC20* and *CCNA2* [89]; *AURKA*, *CHTF18*, *EZH2*, *FBXW7*, *JAG1*, *POP1*, and *PSMB9* [90]; *GBP4*, *OR8K3*, *GABRA2*, *RIPPLY2*, and *TRBV5-7* [91]; *CIRBP* and *INPP5K* [92]; *PARVG*, *SYNE4*, and *CDO1* [93]; and *ELFN1-AS1* and *ZNF132* [94], *SYTL1* [95]; of course, these predictions need to be further experimentally validated; however, their potential as diagnostic biomarkers and prognostic variables is extremely high, especially in the perspective of personalized medicine.

The above-mentioned genes can also be very useful for choosing the most appropriate therapeutic approach. Endocrine therapy, especially high-dose progesterone, is extensively used in estrogen receptor/progesterone receptor (ER/PR)-expressing early-stage EC patients, but some patients show progressive progesterone resistance, and in advanced and/or recurrent cases, this approach is inefficient. It has been shown that the progesterone therapy may be ineffective due to *PR* gene promoter hypermethylation [96] or histone modifications, causing the underexpression—or even the absence—of the receptor (reviewed in [97]). Moreover, *PRA* isoform hypermethylation is significantly higher in metastatic EC [98]. Interestingly, the two main forms of PR, namely PRA and PRB, have different roles in progesterone therapy and show different methylation timings, suggesting that different methylases are involved in the silencing process [99,100]; in addition, *HAND2* methylation also seems to be involved in the progesterone response of EC patients [101].

The role of chromatin modification is also pivotal in the choice of drugs to be administered for chemotherapy. Paclitaxel is a molecule able to disrupt tubulin homeostasis, thus interfering with the mitotic spindle assembly and function. Aberrant methylation of the *CHFR* (*checkpoint with Forkhead and RING finger domains*) gene is a frequent event in carcinogenesis. This checkpoint protein delays the entry of cells into mitosis by diminishing cyclin-dependent kinase 1 (CDK1) activity and stalling cells in the G2 phase. *CHFR* hypermethylation is a frequent event in EC; thus, these cells have an intrinsic resistance to this treatment. Reversing *CHFR* hypermethylation is a possible way to (at least partially) restore paclitaxel sensitivity [102,103]. Similarly, *PGK1* knockdown and HSP90 inhibitor 17-AAG result in down-regulation of DNMTs expression; therefore, up-regulation of DNMTs expression may be involved in PGK1-mediated cisplatin resistance in EC [104]. Finally, Fialkova and collaborators found a relationship between apoptosis resistance and methylation status of three genes (*BCL2L11*, *CIDEB*, and *GADD45A*) in EC [105]. All together, these data suggest performing a deep profiling of the chromatin status of ECs to avoid inefficient and time-consuming approaches.

The situation for histone modification is essentially similar to that of DNA methylation. For example, Huang and collaborators found that SMYD3 (SET and MYND domain-containing protein 3) histone lysine methyltransferase plays a significant role in EC progression by affecting NHEJ DNA repair [106]. Several studies reported the deregulation of both HATs and HDACs in EC [68]; however, they frequently showed contradictory results, suggesting that the complexity of histone modification in EC is far from being fully understood. Notably, this is not surprising, considering the level of complexity of histone modifications (for involved residue, quantity, pattern, and type of modification—see Section 2.3) compared to DNA methylation. In this perspective, the development of single-cell transcriptomics, also in consideration of EC intratumoral heterogeneity [107], will likely be of fundamental help in patients’ molecular characterization and precision medicine [108].

### 2.5. Limitations of Identifying EC Biomarkers Using Chromatin Modifications

Although promising, the application of chromatin modifications as biomarkers in clinical oncology, particularly in EC, presents significant challenges. DNA structural changes are inherently dynamic and context-dependent; their distribution varies not only across different cancer types but also within molecular subtypes of the same tumor. In EC, the epigenetic landscape is influenced by the tumor’s molecular classification—POLE-ultramutated; microsatellite instability-high (MSI-H); copy-number low; and copy-number high subtypes—each exhibiting distinct chromatin signatures. This intra- and inter-tumoral heterogeneity complicates the identification of universally applicable chromatin-based biomarkers. Moreover, chromatin states are modulated by physiological variables such as menstrual cycle, cell cycle phase, differentiation status, and environmental stimuli. These factors introduce biological noise, making it difficult to distinguish disease-specific epigenetic alterations from normal physiological variation. Consequently, the specificity and sensitivity of chromatin modifications as diagnostic or prognostic biomarkers may be significantly compromised.

In addition, technical challenges should also be considered. The techniques used to determine and quantify chromatin modifications are very demanding (Table 4); they require high-quality, often fresh or flash-frozen tissue samples, which are not routinely available in clinical settings. The use of formalin-fixed paraffin-embedded (FFPE) tissues, standard in pathology laboratories, introduces cross-linking artifacts that can compromise chromatin integrity and assay reliability. Additionally, variability in sample preparation protocols, antibody specificity (particularly in ChIP-based assays), sequencing depth, and bioinformatic pipelines contributes to significant inter-laboratory variability. The absence of standardized protocols and quality control measures limits reproducibility and impedes clinical translation.

An additional issue is posed by data interpretation, which may be significantly challenging. The output of these analyses does not allow us to directly identify the transcription factors or cofactors involved in gene regulation. This limits the interpretability of chromatin data, particularly when attempting to link epigenetic changes to oncogenic pathways or therapeutic targets. In EC, frequent mutations in chromatin remodeling complexes such as SWI/SNF further complicate the functional interpretation of chromatin alterations, as the downstream effects on gene expression and tumor behavior remain incompletely understood. To overcome these limitations, a multi-omics approach will be valuable, as it integrates data from different sources (genomic alterations, transcriptomic variations, and proteomic signatures); however, this approach is still computationally intensive and lacks standardized analytical frameworks suitable for clinical applications.

**Figure 3:**
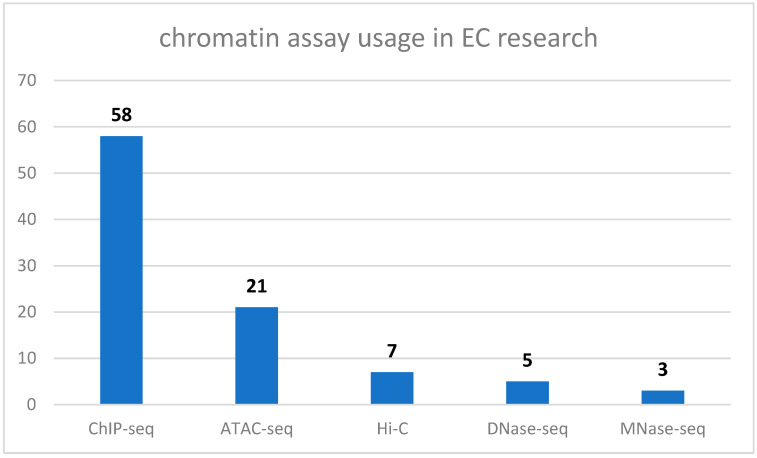
Chromatin assay usage in EC research, according to the NCBI Sequence Read Archive (SRA), and analyzed using artificial intelligence. Numbers over each bar represent the number of studies employing each assay type.

An analysis of chromatin assay usage in EC research is reported in Figure 3, based on simulated metadata from the NCBI Sequence Read Archive (SRA; data retrieved on 22 July 2025; URL: https://www.ncbi.nlm.nih.gov/sra). Notably, ChIP-seq remains the most widely used assay due to its robustness in profiling histone modifications and transcription factor binding. ATAC-seq has gained popularity for its simplicity and low input requirements, especially for single-cell applications. Hi-C and other 3D genome assays are used selectively for mechanistic studies. DNase-seq and MNase-seq are less common due to technical complexity.

### 2.6. Medico-Legal Framework 

Recent discoveries regarding the role of epigenetic modifications in the pathogenesis of endometrial cancer present new and significant challenges from a medico-legal perspective. The increasing complexity in understanding molecular mechanisms, such as DNA methylation and histone modifications, makes constant updating of diagnostic and therapeutic guidelines essential [18,109]. In Italy, the Gelli-Bianco Law (Law 24/2017) [110] introduced the obligation to refer to accredited and regularly updated clinical guidelines, stipulating that these should be revised every two years. However, the process of drafting or updating such guidelines is often lengthy, complex, and, in many cases, inadequate. As in other areas of medicine, the formulation of specific guidelines for innovative fields such as epigenetics remains insufficient or lacking in gynecologic oncology [111].

Failure to apply or misinterpret existing guidelines, or even recommendations of good clinical practice, may compromise the timeliness and accuracy of diagnosis, negatively impacting the patient’s prognosis [112]. This may have serious implications in terms of professional liability. It is therefore essential that healthcare professionals, particularly oncologists and pathologists, receive appropriate and ongoing training in epigenetic analysis techniques and their clinical implications. A lack of continuous education or failure to stay updated on scientific advances can significantly increase the risk of medico-legal disputes, with potentially serious consequences for the clinician and the healthcare facility.

Another critical medico-legal issue is the quality of clinical documentation. As in many other medical specialties, meticulous and detailed recording of diagnostic tests and procedures performed is essential, along with clear and transparent communication with the patient [113]. Discussions regarding the risks, benefits, and limitations of innovative therapeutic approaches, including those based on epigenetics, must be clear, understandable, and properly documented [114].

The dynamic and potentially reversible nature of epigenetic modifications opens new therapeutic horizons but also raises ethical and legal questions, especially concerning clinical trials and informed consent. In this context, emerging technologies, particularly artificial intelligence, represent a significant opportunity [115,116]. AI can substantially aid in the analysis and interpretation of complex epigenetic data, facilitating earlier and more accurate diagnoses, as well as the identification of personalized therapeutic targets [117,118]. In addition, it is worth noting that epigenetic data can reveal sensitive information not just about the individual but also about family members [119]. It can expose environmental exposures, lifestyle choices, and inherited susceptibilities. That in turn gives rise to unique ethical concerns: such data could, for instance, be used by insurers or employers to discriminate based on predicted cancer risk [120]. Questions and challenges also arise when it comes to determining who owns the data and how such information should be stored, especially when shared across research and clinical databases. Access and equitable opportunities may also pose a challenge: advanced epigenetic testing and therapies (e.g., demethylating agents) [121] may not be equally available and covered by national healthcare systems, which could engender healthcare disparities and inequality: wealthier or urban populations may benefit more than underserved groups. That has a bearing on resource allocation as well, for instance, on the decision whether to prioritize costly and unevenly accessible epigenetic therapies over more broadly applicable treatments [122]. In light of all such, the use of such tools requires clear and up-to-date regulations to ensure transparency, clinical validation, and the protection of patients’ sensitive data. Managing these aspects requires scientific rigor, professional responsibility, and strict adherence to ethical and legal principles that safeguard patient rights [117,123,124,125]. From a medicolegal and policy-making perspective, the medicolegal implications of using epigenetics in cancer care are significant and increasingly relevant as epigenetic testing and therapies enter clinical practice [126,127].

Such deeply complex dynamics intersect law, ethics, and medical liability and include both existing and emerging areas of legal risk and regulation. For instance, failure to properly explain epigenetic testing may expose providers to liability for lack of informed consent [128]. Epigenetics, in fact, relies on complex and multilayered science, hence, patients must be adequately informed about the nature of epigenetic markers (modifications, rather than mutations) [129] as well as the clinical limitations or uncertainty of findings and the possibility of incidental or non-actionable results. Existing case-law cat doubt on whether clinicians should inform relatives about hereditary or familial epigenetic risks [130]. In the U.S., Tarasoff and related case law create ambiguity as to whether a physician have a duty to warn third parties (e.g., family members) of inherited cancer risk [131]. Since epigenetic risks may not always be straightforwardly heritable, a further element of complication emerges. The sensitive nature of data management is also noteworthy: unauthorized access or use of epigenetic data may violate laws such as the Health Insurance Portability and Accountability Act (HIPAA) in the U.S. and the General Data Protection Regulation (GDPR) in Europe [132]. Still, in the U.S., the Genetic Information Nondiscrimination Act (GINA) does not explicitly cover epigenetics.

## 3. Conclusions

The human endometrium is a highly dynamic tissue that undergoes cyclical morphological changes strictly connected to women’s fertility. These changes are mainly driven by hormonal fluxes of estradiol and progesterone. The different phases of the healthy menstrual cycle are characterized, at the molecular level, by distinct patterns of gene expression, which are regulated in part by epigenetic mechanisms such as DNA methylation, post-translational histone modifications, and the action of non-coding RNAs [133]. These mechanisms make understanding pathological endometrium a challenge. The complexity of the epigenetic gene expression control in EC through competing endogenous RNA networks (ceRNETs) has been recently discussed [134]. The complexity of epigenetic mechanisms acting through chromatin modifications is equally intricate. The endometrium, being a dynamic structure, is crucial to fully understand its changes over time, not only during the menstrual cycle but also in relation to women’s age. Understanding these sometimes-subtle differences in every phase of a woman’s life is the main challenge to efficiently approaching EC.

Clinically, understanding chromatin modifications in endometrial cancer may improve patient stratification, support personalized fertility-sparing approaches, and inform the development of new targeted therapies.

## Figures and Tables

**Table 1:** Cancer driver genes involved in chromatin modification, as reported in [40] for both gene name and impaired pathway. Data ordered according to gene name. Asterisks mark genes whose expression is also impaired in UCEC (uterine corpus endometrial carcinoma).

Gene Name	Pathway
*AJUBA*	chromatin other
ARID1A*	chromatin SWI/SNF complex
*ARID2*	chromatin SWI/SNF complex
ARID5B*	chromatin histone modifiers
*ASXL1*	chromatin other
*ASXL2*	chromatin other
ATF7IP*	chromatin other
*ATRX*	chromatin SWI/SNF complex
BCOR*	chromatin other
*BRD7*	chromatin SWI/SNF complex
*CHD3*	chromatin other
CHD4*	chromatin other
CHD8*	chromatin other
*CREBBP*	chromatin histone modifiers
CTCF*	chromatin other
*DNMT3A*	epigenetic DNA modifiers
EP300*	chromatin histone modifiers
*H3F3A*	chromatin other
*H3F3C*	chromatin other
*HIST1H1E*	chromatin other
KANSL1*	chromatin histone modifiers
*KDM5C*	chromatin histone modifiers
*KDM6A*	histone modification
*KMT2A*	chromatin histone modifiers
KMT2B*	chromatin histone modifiers
KMT2C*	chromatin histone modifiers
*KMT2D*	chromatin histone modifiers
*MEN1*	chromatin histone modifiers
*NCOR1*	chromatin histone modifiers
*NIPBL*	chromatin other
*NPM1*	chromatin other
*NSD1*	chromatin histone modifiers
PBRM1*	chromatin SWI/SNF complex
*PPP6C*	histone modification
*SETD2*	histone modification
SIN3A*	chromatin histone modifiers
*SMARCA1*	chromatin SWI/SNF complex
*SMARCA4*	chromatin SWI/SNF complex
*SMARCB1*	chromatin SWI/SNF complex
*WHSC1*	chromatin histone modifiers
*ZMYM3*	chromatin histone modifiers

**Table 2:** Proteins involved in chromatin modification. Proteins acting on DNA structure and compaction can be generally divided into writers, readers, and erasers. These names are assigned to both DNA and histone modifiers [18].

Type	Action	Examples *
Writers	Addition of chemical groups to DNA or histones	DNMT, HAT, KMT, PRMT
Readers	Modification recognition and modifiers recruiting	Chromo/bromo-domains
Erasers	Removal of chemical groups from DNA or histones	HDAC, KDM

* Abbreviations: DNMT: DNA methyltransferase; HAT: Histone acetyltransferase; KMT: Lysine methyltransferase; PRMT: Protein arginine methyltransferase; HDAC: Histone deacetylase; KDM: Lysine demethylase.

**Table 3:** Examples of chromatin remodeling genes frequently mutated in EC.

Gene	Function	Clinical Relevance in EC
*ARID1A*	Component of SWI/SNF complex; chromatin remodeling	Frequently mutated; associated with poor prognosis
*SMARCA4*	ATPase subunit of SWI/SNF complex	Loss linked to dedifferentiated tumors
*CHD4*	Part of NuRD complex; transcriptional repression	Mutations associated with microsatellite instability
*EZH2*	Histone methyltransferase; H3K27me3 writer	Overexpression linked to aggressive tumor behavior
*KMT2D*	Histone methyltransferase; H3K4 methylation	Mutations observed in endometrioid subtypes

**Table 4:** Chromatin assay methods and their limitations.

Assay Type	Description	Use	Limitations
ChIP-seq	Chromatin immunoprecipitation followed by sequencing	Identifies DNA regions bound by specific proteins	Requires high-quality antibodies; low resolution; poor reproducibility; not suitable for FFPE samples
ATAC-seq	Assay for transposase-accessible chromatin using sequencing	Measures chromatin accessibility	Sensitive to sample quality; limited in FFPE; limited resolution in complex tissues
DNase-seq	DNase I hypersensitive site sequencing	Maps DNase I hypersensitive sites	Requires large input material; complex protocol; fresh tissue; low throughput
MNase-seq	Micrococcal nuclease digestion followed by sequencing	Profiles nucleosome positioning	Bias toward accessible regions; digestion variability; complex data interpretation
Hi-C	Measures the frequency at which two DNA fragments physically associate in 3D space through crosslinking	Captures 3D chromatin interactions	High cost; complex data interpretation
CUT&RUN	Antibody-targeted controlled cleavage by micrococcal nuclease followed by massive parallel DNA sequencing	Analyzes DNA/protein interactions	Emerging method; limited clinical validation

## Data Availability

The original contributions presented in this study are included in the article. Further inquiries can be directed to the corresponding author.
